# Cancer Stem Cells and the Slow Cycling Phenotype: How to Cut the Gordian Knot Driving Resistance to Therapy in Melanoma

**DOI:** 10.3390/cancers12113368

**Published:** 2020-11-13

**Authors:** Luigi Fattore, Rita Mancini, Gennaro Ciliberto

**Affiliations:** 1 Department of Research, Advanced Diagnostics and Technological Innovation, SAFU Laboratory, Translational Research Area, IRCCS Regina Elena National Cancer Institute, 00144 Rome, Italy; luigi.fattore@ifo.gov.it; 2 Department of Melanoma, Cancer Immunotherapy and Development Therapeutics, Istituto Nazionale Tumori IRCCS, “Fondazione G. Pascale”, 80131 Naples, Italy; 3 Department of Clinical and Molecular Medicine, Sant’ Andrea Hospital, Sapienza University of Rome, 00161 Rome, Italy; rita.mancini@uniroma1.it; 4 Scientific Directorate, IRCSS Regina Elena National Cancer Institute, 00144 Rome, Italy

**Keywords:** melanoma, target therapy, cancer stem cells, slow cycling phenotype, drug resistance, OXPHOS, lipid metabolism

## Abstract

**Simple Summary:**

Cancer stem cells play a central role in the development of cancer and are poorly sensitive to standard chemotherapy and radiotherapy. Furthermore, they are also responsible for the onset of drug resistance. This also occurs in malignant melanoma, the deadliest form of skin cancer. Hence, cancer stem cells eradication is one of the main challenges for medical oncology. Here, we conducted a bioinformatics approach aimed to identify the main circuits and proteins underpinning cancer stem cell fitness in melanoma. Several lessons emerged from our work and may help to conceptualize future therapeutic approaches to prolong the efficacy of current therapies.

**Abstract:**

Cancer stem cells (CSCs) have historically been defined as slow cycling elements that are able to differentiate into mature cells but without dedifferentiation in the opposite direction. Thanks to advances in genomic and non-genomic technologies, the CSC theory has more recently been reconsidered in a dynamic manner according to a “phenotype switching” plastic model. Transcriptional reprogramming rewires this plasticity and enables heterogeneous tumors to influence cancer progression and to adapt themselves to drug exposure by selecting a subpopulation of slow cycling cells, similar in nature to the originally defined CSCs. This model has been conceptualized for malignant melanoma tailored to explain resistance to target therapies. Here, we conducted a bioinformatics analysis of available data directed to the identification of the molecular pathways sustaining slow cycling melanoma stem cells. Using this approach, we identified a signature of 25 genes that were assigned to four major clusters, namely (1) kinases and metabolic changes, (2) melanoma-associated proteins, (3) Hippo pathway and (4) slow cycling/CSCs factors. Furthermore, we show how a protein−protein interaction network may be the main driver of these melanoma cell subpopulations. Finally, mining The Cancer Genome Atlas (TCGA) data we evaluated the expression levels of this signature in the four melanoma mutational subtypes. The concomitant alteration of these genes correlates with the worst overall survival (OS) for melanoma patients harboring BRAF-mutations. All together these results underscore the potentiality to target this signature to selectively kill CSCs and to achieve disease control in melanoma.

## 1. Introduction

Melanoma remains one of the most widespread types of cancer in western countries, and its incidence is rapidly increasing [1,2]. In recent years, immunotherapy and targeted therapies have changed the treatment scenario for advanced melanoma [3,4,5,6].

Selective inhibitors of V600E BRAF-mutated melanoma, such as vemurafenib, dabrafenib and encorafenib, prolong survival of patients harboring the V600E mutation [3,4,7,8]. However, the onset of tumor resistance observed following this treatment, which was found to be related to the emergence of bypass mutations in resistant tumors that often cause reactivation of the RAS/BRAF/MEK pathway [9,10], led to the development of combo therapies with BRAF and MEK inhibitors as the current standard of care [11,12,13]. Unfortunately, dual therapy, although being able to provide more durable disease control compared to BRAF inhibitors alone, is also plagued by the development of drug resistance [14,15].

Beside genetic mechanisms, a plethora of non-genetic changes have been identified to be involved in the evolution of resistance to target therapy in melanoma [16,17,18], the main ones being: (1) the induction of changes in the inflammatory niche driving drug tolerance [19]; (2) the displacement of the bioenergetic equilibrium [20] and (3) the involvement of receptor tyrosine kinases (RTKs). As for the last mechanisms, for example, our group and others have demonstrated a key role of ErbB3 receptor up-regulation upon exposure to BRAF and MEK inhibitors [21,22]. In addition, also non-coding RNAs, such as microRNAs are emerging as key players able to orchestrate epigenetic and non-genomic mechanisms of resistance to target therapy in melanoma [23,24,25,26,27,28].

Similarly, multiple reports document the development of resistance to immune checkpoint inhibitors [29,30,31]. Therefore, strategies aimed to reduce the onset of resistance are of the utmost importance in the therapy of melanoma [31].

Cancer stem cells (CSCs) are known to be involved in the development of resistance to treatment, thereby contributing to disease relapse after an initial response [32,33,34,35]. CSCs, also known as tumor-initiating cells, are cells that can perpetuate themselves by self-renewal [36], and present peculiar characteristics, including the expression of specific surface markers [34,37]. The theory of CSCs has originally been described as a bone fide biological phenomenon in hematologic tumors, such as leukemia. From here, they have also been identified in solid malignancies, such as lung, breast and colon cancers. Differently, in the case of melanoma, some debates on the existence of CSCs are still ongoing mostly due to the difficulty to identify reliable markers for their identification [38].

CSCs are clearly linked to tumor heterogeneity, which is also a hallmark of cancer development and at the basis of resistance to anti-neoplastic treatments [39]. Heterogeneity is observed at different levels, for example, within a single tumor (intra-tumoral) or between tumor masses of the same histopathological subtype in the same patient (inter-tumoral) or between tumors of the same histotype deriving from different patients (inter-patient) [40,41]. Moreover, heterogeneity can occur at a spatial level (uneven distribution of genetically and/or epigenetically different subpopulations within the same or synchronous lesions in the same patient), or at a temporal level (i.e., dynamic variations of tumor cells over time) [39,41]. Notably, temporal heterogeneity may also encompass the existence of a tiny pre-existing subpopulation of cells, which emerge as the dominant population under the pressure imposed by a given therapy. Tumor heterogeneity can be influenced in response to the selective pressure of the immune system or of antineoplastic treatments [42].

According to the CSC model, tumors are per se heterogeneous and are organized in a hierarchical manner [43]. At the top of the hierarchy is a small fraction of cells called CSCs, which are endowed with the ability to undergo both symmetrical and asymmetrical divisions. These cells can differentiate into “non-CSCs”, with the acquisition of stable genetic and/or epigenetic changes [44]. According to the model, non-CSCs represent the largest fraction of the tumor. Nowadays the alternative models postulated to explain tumor evolution are: linear, branching, neutral and punctuated [45]. According to the first model tumor cells acquire mutations linearly in a step-wise process leading to more malignant stages of cancer. The branching model predicts that single clones originate from a common ancestor, and evolve simultaneously in the tumor mass because of their increased fitness. Neutral evolution is a case of branching evolution for which no selection or fitness changes occur during the lifetime of the tumor and influence the evolution of the clones. Finally, and differently from the other models, the theory of “punctuate equilibrium” assumes that mutations are not acquired gradually and sequentially over time but in short bursts of tumor progression. According to this model, intratumor heterogeneity is higher in the early stages in which one or a few dominant clones stably expand to form the bulk of tumor mass. Interestingly, a recent mathematical modeling has revealed that bursts of mutations are the best models able to recapitulate the long-stemmed clonal trees of the evolution of different cancers. For further details we recommend several excellent reviews on this topic such as the work by Davis and colleagues [45].

More recently, those models have been challenged by several pieces of evidence showing a plastic interconversion of epigenetic changes underpinning stem phenotype. Thereby, the theory of CSCs has been reconciled with the evidence of a dynamic interplay between slow cycling (and drug-resistant) and fast cycling (drug-sensitive) states [46]. Although the correspondence of CSCs with slow cycling cells within a tumor is still debated, it is widely accepted that tumor heterogeneity is linked to a slow cycling and plastic sub-population of cells able to challenge therapeutic efforts and to emerge as drug-resistant survivors.

In this context, the “phenotype switching” model has been proposed [47]. It has been conceptualized for malignant melanoma, which is notorious for the high level of genetic and non-genetic heterogeneity [48,49]. Therefore, it is thought that melanoma cells have the possibility to shift between different transcriptional programs mostly depending on the oscillation of the microphthalmia-associated transcription factor MITF [46]. These states are the “proliferative/differentiative” or MITF^high^ and the “invasive” or MITF^low^ (also known as rheostat model) [50]. Furthermore, molecular changes leading to tumor heterogeneity are also regulated by local microenvironment cues (e.g., interactions with non-tumor cells, hypoxia, stroma-derived factors HGF, TGF-β) [47]. For example, it has been recently reported that an acidic tumor microenvironment influences a stem-like phenotype in melanoma [51]. According to “phenotype switching” model, non-hierarchical plasticity may lead to the transient existence of slow-cycling cells able to survive to therapeutic pressures activating compensatory signaling pathways. Thereby, it has been named “dynamic stemness”. Those aspects and their therapeutic implications will be discussed later in our review. Noteworthily, those melanoma cell subpopulations possess neural crest stem cell features and are dedifferentiated further than the canonical invasive phenotype [52,53].

Coherently, one of the main markers of this state, namely the nerve growth factor receptor (NGFR) has been described as a putative melanoma CSC marker [54].

Along the same topic, H3K4 demethylase JARID1B has been identified as a marker of slow cycling cells in melanoma. According to the notion of CSCs, the subpopulation of cells express this protein cycle very slowly (times of >4 weeks) compared to the rest of the rapidly proliferating main population [55].

Recently, the development of single cell (sc) analysis approaches, which are able to better characterize tumor heterogeneity and CSCs have represented a major technological breakthrough [42]. For example, sc-RNAseq studies have confirmed the presence of a small population of non-cycling cells in both melanoma cell lines in vitro, as well as from those derived from freshly processed melanomas. Coherently, those cells are enriched for CSC markers like JARID1B and NGFR [56,57].

In this paper we have tried to interpret available data using a bioinformatic approach directed to identify novel targets to selectively hit CSCs in BRAF mutated cutaneous melanomas. This led us to identify a common interaction network encompassing the existence of four major clusters which may be at the basis of CSC fitness. Importantly, the signature of genes belonging to this network showed a prognostic potential for BRAF-mutant melanoma patients based on The Cancer Genome Atlas (TCGA) data. Finally, we discuss how to potentially tackle it at multiple levels to selectively block the spread of those cells and to prolong the efficacy of target therapy in melanoma.

## 2. Results

### 2.1. Identifying Common Denominators for Melanoma Stem Cell Survival through Bibliographic Search

CSCs are charged to be among the main determinants of the failure of anticancer treatments. This is also the case of target therapy in *BRAF*-mutant melanomas. An open question remains whether common molecular pathways sustain slow cycling cells under selective pressure by therapeutic agents and foster their growth as drug-resistant survivors. In this regard, there is a general agreement to consider metabolic rewiring as a central process responsible for promoting CSC fitness [20]. Notwithstanding the involvement of these common pathways, the specific players identified differ among different studies.

**Figure 1:**
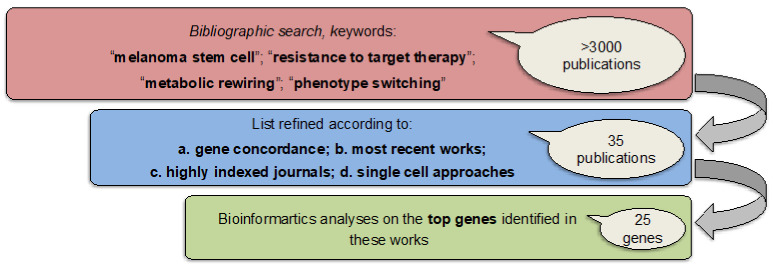
Schematic workflow of the study divided into three steps to obtain a list of 25 top genes for bioinformatics analyses.

In order to identify common denominators, we carried out the following effort in three steps (Figure 1).

(1) We first conducted an extensive bibliographic search using as keywords “melanoma stem cells”, “resistance to target therapy”, “metabolic rewiring” and “phenotype switching” and we found more than 3000 publications. (2) We refined this list according to the: (a) potential concordance in the genes identified among studies, i.e., for their presence in at least two of them (b) most recent works (c) highly indexed journals (impact factor >7) and (d) studies taking advantages of single-cell approaches. These parameters allowed us to restrict the list of publication up to 35. (3) These publications were screened to finally obtain a list of 25 top genes, which potentially sustain melanoma stem cell fitness. In detail, the complete list is available as Table 1. This signature will be named from now simply “MSCsign”.

Here below we recapitulate the main findings of the major studies emerging from our analysis. Rambow et al. have reported that BRAF/MEK inhibition enriches multiple therapy-resistant slow cycling populations, which retain the ability to proliferate in the absence of therapeutic stress [53]. Those cells exhibited neural crest stem cell transcriptional programs largely driven by the nuclear receptor RXRG. Another mechanism proposed to support the onset of drug resistance is the epigenetic reprogramming induced by therapy [58]. Reprogramming may be initiated with a loss of SOX10-mediated differentiation, and then followed by a multi-stage process involving the activation of new signaling pathways, such as Jun-AP-1 and TEAD [58]. These events lead to post-treatment transition to stable resistance phenotypes characterized by well-known markers, such as the receptor tyrosine kinase AXL. Furthermore, it has been reported in in vivo mouse models that slow cycling melanoma cells which adaptively resist to BRAF/MEK inhibitors (MAPKi) are also capable of reentering the cell cycle and give rise to highly metastatic subclones that invade different tissues [70]. Importantly, these cells show a dedifferentiated state characterized by high levels of cancer stem cell markers, such as NGFR and JARID1B. Notably, all these studies converge to support the notion that drug treatment initially induces a fast increase in de-differentiation toward a slow cycling CSC state characterized by high NFGR levels [53,66]. In essence, the neural crest stem cell signature could be defined as “point of entry” for the development of resistance to target therapy in melanoma [71].

While CSCs are poorly sensitive to chemotherapy and target therapies that mainly act by blocking cell cycle, several studies have shown that they are sensitive to the interference with signaling pathways [68,73]. Zakaria et al. have suggested that NF-κB inhibition reduces the ability of CSCs to maintain their population within the tumor mass [73]. Along the same line, Su and colleagues have recently demonstrated that the switch from rapidly dividing drug-responsive to drug-tolerant/slow cycling states early occurs upon exposure to MAPKi in melanoma. This event mostly encompasses the activation of NF-κB. Coherently, its inhibition together with MAPKi keeps melanoma cells in a drug-sensitive state [66].

In a similar study, the same result was achieved by modulating the p53, NF-κB and HIF-1α pathways [74]. Paradoxically, inhibition of TP53 was shown to sensitize melanoma cells to BRAF/MEK inhibition [69]. In addition, SerpinE2 appears to be involved in the maintenance and invasiveness of CSCs in melanoma, and the inhibition of this proteolytic enzyme has been speculated to be a potential therapeutic target [68].

As previously stated, the alteration of the metabolic status is a hallmark of CSC maintenance and mostly encompasses: (1) increased oxidative phosphorylation (OXPHOS) and (2) lipidome alterations.

As to the first, it is widely accepted that therapy resistant slow-cycling melanoma cells are addicted to mitochondrial OXPHOS: this vulnerability represents a source of therapeutic opportunities [75]. Roesch et al. firstly described through proteome profiling, which the JARID1B^high^ subpopulation of cells upregulate several enzymes involved in mitochondrial oxidative ATP synthesis [55,72]. Coherently, inhibition of mitochondrial respiration (using oligomycin, rotenone and phenformin) blocked the emergence of these slow cycling cells and potentiated the tumor-suppressive potential of BRAFi in vivo. In a subsequent study, the same group demonstrated that MAPKi induce the upregulation of mitochondrial biogenesis in intrinsically resistant melanoma cells [65]. The authors found an increase in mitochondrial DNA copy number, mitochondrial mass, maximal oxygen consumption rate, and reactive oxygen species production in these cells upon exposure to the drugs. In contrast, therapy sensitive cells show the opposite pattern, i.e., they downregulated transcriptional signatures associated with MitoBiogenesis. Therefore, the specific mitochondrial HSP90 inhibitor, namely Gamitrinib was demonstrated to be effective in eradicating intrinsically resistant cells and increased the efficacy of MAPKi in vitro and in vivo [65].

Along the same topic, Vazquez et al. have demonstrated the existence of an additional adaptive metabolic program in melanoma that is dependent upon the translocation to the nucleus of the master regulator of melanocyte MITF, which, in turn, activates PGC1α, a key regulator of mitochondrial respiration [61]. Mechanistically, MITF^high^/PGC1α^high^ cells exhibit increased OXPHOS coupled with ROS detoxification capacities enabling them to survive under oxidative stress conditions [61]. Hence, it is not surprising that the mTORC1/2 inhibitor AZD8055, which triggers MITF cytoplasmic retention, was able to decrease PGC1α expression and OXPHOS in melanoma [62]. Thereby, this compound potentiated the efficacy of MAPKi in BRAF-mutated melanoma cells in vitro and in vivo [62]. In line with this finding the novel mitochondrial complex I inhibitor IACS-010759 demonstrated a significant anti-tumor activity as single-agent of in high OXPHOS MAPKi-resistant melanoma models in vivo [67].

Noteworthy, the existence of JARID1B^high^/PGC1α^high^ cells has been reported in melanoma according to their relevance in sustaining high-OXPHOS metabolism [63].

CSCs maintenance also seems to depend upon the size of the pool of monounsaturated fatty acids (MUFAs) generated by the activity of the stearoyl-CoA desaturase 1 (SCD1) [37,76,77,78] because SCD1 inhibition was shown to selectively eliminate CSCs in lung cancer, both alone and in synergy with chemotherapy [79,80].

Pisanu et al. investigated the role of SCD1 and its inhibition by a specific compound, MF-438, in melanoma CSCs, by a comprehensive approach employing bioinformatics and 2D and 3D cultures [64]. In line with the initial hypothesis of the importance of SCD1 in maintaining the CSCs pool in melanoma, the expression of this gene increased during melanoma progression. Moreover, *BRAF*-mutated melanoma cell cultures enriched in CSCs showed an overexpression of SCD1 and were more resistant to BRAF and MEK inhibitors than non-enriched cultures. Exposure of *BRAF*-mutated melanoma cells to inhibitors of the MAPK pathway enhanced stemness features by increasing the expression of YAP/TAZ and downstream genes, but not SCD1. However, the pharmacological inhibition of SCD1 by MF-438 downregulated YAP/TAZ and was able to revert CSC enrichment and resistance to MAPK inhibitors [64]. These findings, albeit limited to in vitro studies, underscore the potential role of SCD1 in melanoma progression and suggest the opportunity to further SCD1 inhibitors in combination with MAPK inhibitors for the control of resistance to targeted therapy.

Very recently, Vivas-Garcia et al. investigated the impact of the tumor microenvironment on phenotype switching focusing on fatty acid metabolism [60]. The authors observed the downregulation of both MITF and SCD in melanoma cells following glutamine deprivation with an alteration of the balance of saturated fatty acids and MUFAs [60]. As expected, MITF was accompanied by a differentiation/proliferative-to-invasive switch characterized also by hallmarks of drug resistance (i.e., AXL). However, in apparent contrast with Pisanu et al., in this model, loss of SCD1 was accompanied by an increase in cells with an invasive phenotype. Further studies are needed to deepen understanding of the role of SCD1 in melanoma, for example, taking advantage of single cell approaches.

In addition, the metabolism of fatty acid oxidation (FAO) has been reported to govern stem cell balance between quiescent and proliferative states [81]. In this context, Aloia et al. have recently demonstrated that an FAO metabolic shift early occurs in BRAF-mutant melanoma cells upon exposure to MAPKi [59]. Mechanically, this is characterized by the upregulation of peroxisome proliferator-activated receptor α (PPARα) and carnitine palmitoyltransferase 1A (CPT1A) enzymes. Coherently, the upfront inhibition of FAO and MAPK synergistically inhibits tumor cell growth in vitro and in vivo [59].

### 2.2. Identification of a Common Interactome Sustaining Melanoma Stem Cell Fitness Divided into Four Major Clusters

First of all we subjected the 25 genes of “MSCsign” to Markov Cluster Algorithm for bioinformatics clustering based on protein−protein interaction (PPI) and similarity networks [82]. This led to the identification of four major clusters (represented in Table 1). On the basis of the genes present in these clusters, these signatures were named: (1) kinase and metabolic, (2) melanoma-associated, (3) Hippo pathway and (4) slow cycling/CSCs. The first cluster includes nearly 50% of the genes (i.e., 12 out of 25). Among them, there are BRAF, MEK, AKT kinases as well as metabolic enzymes, such as SCD, CPT1A and PPARGC1A. The melanoma-associated cluster includes lineage specific genes, such as MITF and MLANA, as well as markers associated with resistance to target therapy in melanoma, such as AXL and NFKB. The third cluster encompasses three genes all belonging to the Hippo oncogenic signaling (i.e., YAP, TAZ and TEAD). Finally, the last one includes NGFR and KDM5B (or JARID1B). For this reason, it was named the slow cycling/CSCs cluster.

**Figure 2:**
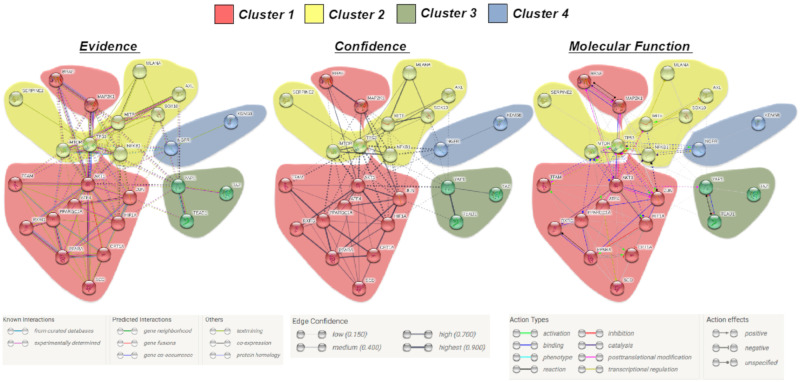
Evidence, confidence and molecular function-based protein−protein interaction (PPI) networks performed using the list of 25 top genes relevant for melanoma cancer stem cells (CSCs) and resistance to target therapy. The legends indicate the meaning of the lines. Interaction score applied >0.4 (medium confidence). A total of 89 were hedges obtained with a PPI enrichment *p* < 1.0 × 10^−16^. https://string-db.org.

In the next step, we used “MSCsign” to build a PPI network using the STRING online database [83]. This software allows us to complement available information of PPI with computational predictions to generate a global network including direct (physical), as well as indirect (functional) interactions. Using a minimum required interaction score (>0.4, medium confidence) we plotted the interactome of the 25 genes as connected by 89 hedges with a PPI enrichment *p* < 1.0 × 10^−16^. The displayed networks available are based on: (1) evidence, as multiple lines where the color indicates the type of interaction; (2) confidence, where line thickness denotes the strength of data support and (3) molecular action, in which different lines represent the predicted mode of action (Figure 2). Again, we obtained four clusters with a partial overlap between signatures 1 and 2, i.e., the kinase/metabolic and melanoma-associated, respectively. Differently, the hippo and slow cycling/CSCs clusters segregated separately from all the others. Furthermore, the same list was also subjected to gene-set enrichment analyses using well-known classification systems, such as Gene Ontology, KEGG (Kyoto Encyclopedia of Genes and Genomes) and Reactome (Table S1). Notwithstanding the different clustering, all PPI networks converge in demonstrating that these 25 genes are closely interconnected (Figure 2). This suggests that a synchronous interactomic profile may exist and can be potentially tackled as will be discussed later.

### 2.3. TCGA Data Mining Uncovers the Prognostic Value of “MSCsign” in BRAF-Mutant Melanomas

**Figure 3:**
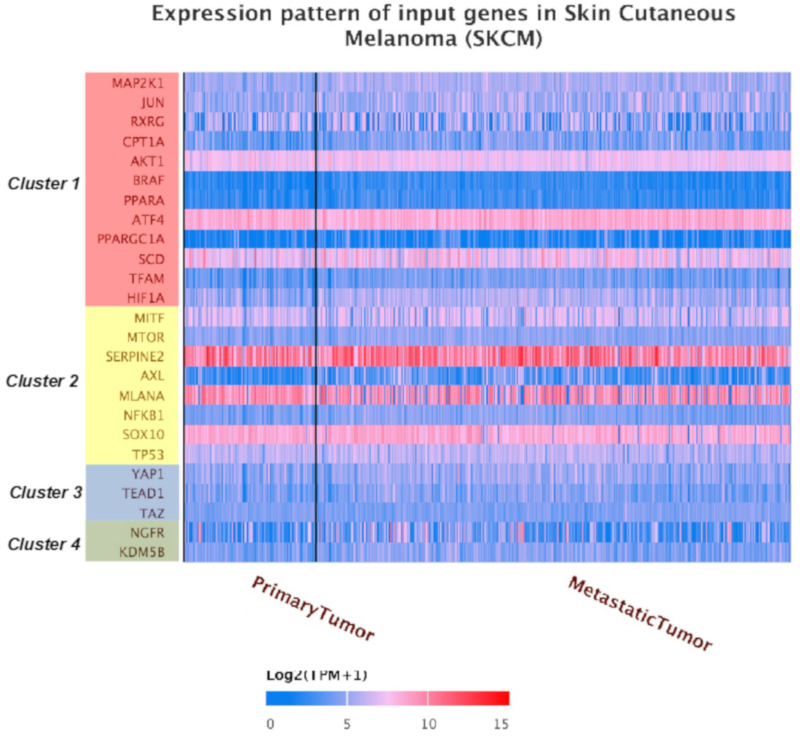
Expression levels of the 25 top genes relevant for melanoma CSCs and resistance to target therapy on 480 samples of skin cutaneous melanoma from The Cancer Genome Atlas. http://ualcan.path.uab.edu/index.html.

Next, we addressed the potential prognostic value of the “MSCsign” for skin cutaneous melanoma patients. Towards this goal one of the most reliable methods is The Cancer Genome Atlas (TCGA) data mining. Hence, first of all we checked the expression levels of the aforementioned 25 genes in 480 samples of skin cutaneous melanoma using UALCAN software [84]. The results underscore the high levels of heterogeneity of gene expression among the different melanoma specimens, as already well-known for this tumor [41]. Coherently, the most expressed markers belong to cluster 2 according to its melanoma-associated signature (Figure 3). It is important to point out that certain genes especially those belonging to cluster 3 and 4 are expressed at low levels. These data probably reflect a pre-therapy scenario and the activation of specific clusters of genes might occur following treatment in order to escape from the therapeutic pressure. Notably, gene expression levels were also normalized considering the maximum median expression value across all the blocks (Table S2).

Thereafter, we decided to investigate the biological meaningfulness of “MSCsign” stratifying melanoma patients according to the mutational subtype again through TCGA data. To this aim we used the skin cutaneous melanoma dataset (TCGA, PanCancer Atlas) [85] available on cBioPortal website [86,87]. In detail, this contains data of 448 melanoma samples of which 440 were profiled for the mutational status and 443 further subjected to RNA-Seq analyses.

**Figure 4:**
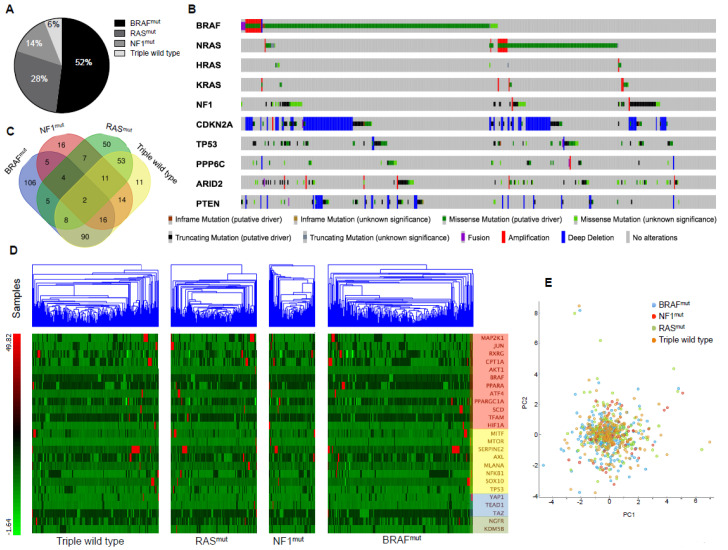
Melanoma stem cell signature (“MSCsign”) expression levels evaluated according to skin cutaneous melanoma mutational subtypes. (**A**) Cake graph representing the most common mutational subsets of metastatic melanoma. (**B**) OncoPrint evaluation of distinct genomic alterations. (**C**) Venn diagrams showing the overlapping of the patients belonging to the four mutational subtypes. (**D**) Heat-maps of the expression levels of the 25 genes of “MSCsign” clustered according to the mutational subsets. (**E**) Principal component analysis performed on the expression levels of “MSCsign” in the four mutational subtypes. Data from https://www.cbioportal.org/.

According to the genomic classification and mutational status [85] this tumor is categorized into four main molecular subtypes: BRAF^mut^, RAS^mut^, NF1^mut^, or triple wild type. The incidence of these mutational states [10,85] is represented as a cake graph in Figure 4A. It is important to point out that the effects of the three most common driver mutations (BRAF^mut^, RAS^mut^, NF1^mut^) are influenced by additional mutations in other genes, such as CDKN2A and PTEN [10]. This was confirmed by the OncoPrint evaluation of melanoma genomic alterations obtained through the cBioPortal online tool (Figure 4B). Following this approach, we extracted gene expression data of “MSCsign” of 237 samples from BRAF^mut^, 140 from RAS^mut^, 76 from NF1^mut^ and 205 from triple wild type subtypes. This latter subtype is the most overlapping to the others as evident by the Venn Diagrams shown in Figure 4C. These data were then subjected to hierarchical clustering to investigate whether a specific melanoma subtype might particularly express one of the four clusters of “MSCsign”. Results shown as heat-maps in Figure 4D demonstrate that the expression levels of the 25 genes are similar among the four mutational subtypes. These findings were confirmed by principal component analyses which demonstrated that the “MSCsign” is not able to distinguish a specific mutational subtype from the others (Figure 4E). The raw data relative to gene expression levels are reported in Table S3.

**Figure 5:**
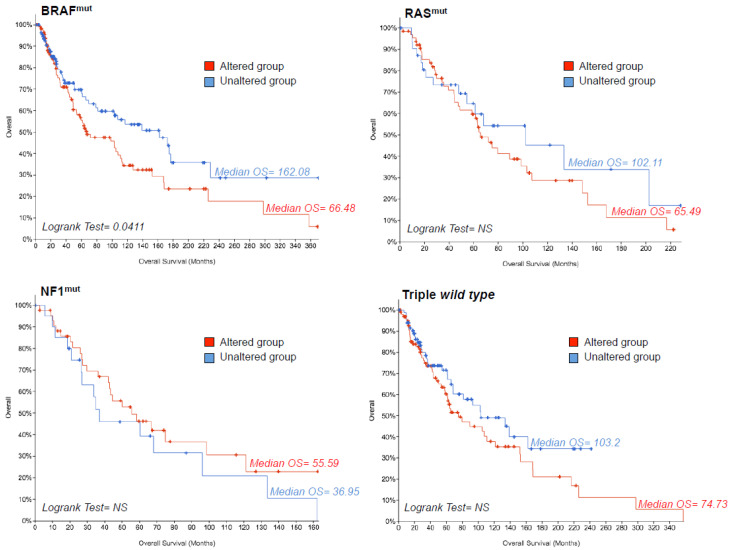
Kaplan–Meyer curves evaluating the prognostic value of “MSCsign” in the four mutational subtypes of skin cutaneous melanoma. Data plotted from https://www.cbioportal.org/.

Finally, we decided to investigate the prognostic potential of “MSCsign” in the aforementioned mutational subtypes. Our results shown as Kaplan–Meyer curves demonstrated that the alteration of the 25 genes is statistically correlated with the worst overall survival (OS) only in the specific subset of BRAF^mut^ melanoma patients (Figure 5). All data relative to OS are available as Table S4. Again, these findings suggest the possibility that BRAF^mut^ melanomas tend to activate specific clusters of genes in order to escape treatments.

**Figure 6:**
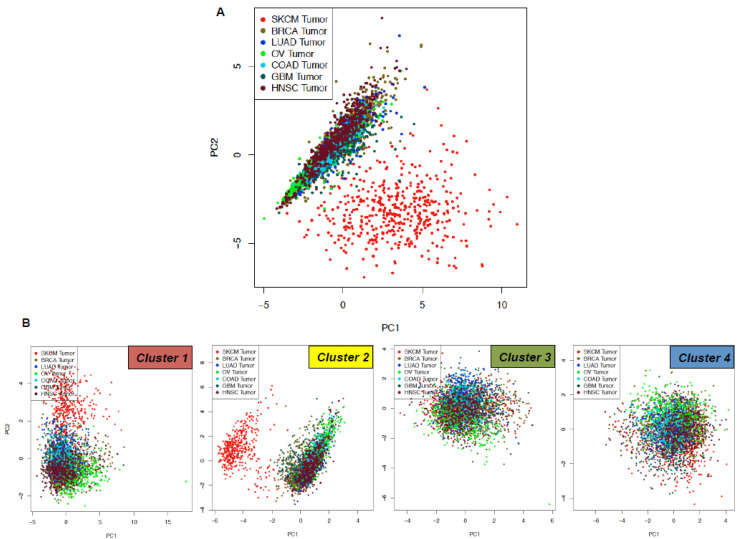
Principal component analyses performed using the expression levels of (**A**) all the 25 genes of “MSCsign” based on The Cancer Genome Atlas (TCGA) data and (**B**) divided according to the four different clusters identified. SKCM = Skin Cutaneous Melanoma, BRCA = Breast invasive carcinoma, LUAD = Lung adenocarcinoma, OV = Ovarian serous cystadenocarcinoma, COAD = colon adenocarcinoma, GBM = Glioblastoma multiforme, HNSC = Head and Neck squamous cell carcinoma. http://gepia.cancer-pku.cn/index.html.

Hereafter, we performed a principal component analysis using the expression levels of “MSCsign” from the skin cutaneous melanoma dataset in comparison with those of other six solid tumors (all data coming from The Cancer Genome Atlas). They are: breast invasive carcinoma, lung adenocarcinoma, ovarian serous cystadenocarcinoma, colon adenocarcinoma, glioblastoma multiforme and head and neck squamous cell carcinoma. Principal component analysis results show that our molecular signature distinguishes melanomas from all the other tumors (Figure 6A). Data were plotted through the online software GEPIA [88]. Finally, we further refined the principal component analyses to the single clusters belonging to “MSCsign”. Results shown in Figure 4B clearly demonstrate that cluster 2 genes are the only ones able to fully distinguish cutaneous melanomas from all the other tumors. This is coherent with the “melanoma-associated” feature of this signature. Differently, the other three clusters of “MSCsign” commonly are segregated in the graphs for all the seven solid tumors tested (Figure 6B).

## 3. Conclusions

Drug resistance virtually frustrates every kind of anti-neoplastic treatment. A paradigmatic example is the development of resistance to MAPKi in *BRAF*-mutant melanomas. This phenomenon is largely driven by the selection of cells with a slow cycling phenotype (which can alternatively be called CSCs), which have therefore emerged as the key therapeutic targets for intervention.

In the present work we carried out a novel approach which allowed to identify a signature of genes relevant for melanoma stem cell fitness, namely “MSCsign”. Those genes are correlated to each other in a complex interactome and organized into four different clusters. We postulate that the simultaneous inhibition of multiple effectors belonging to the aforementioned four clusters may be a successful strategy to eradicate CSCs molecular roots. This hypothesis is supported by the finding that alterations of the expression of genes belonging to “MSCsign” is associated with the worst OS for BRAF mutated melanoma patients based on TCGA data.

Metabolic rewiring is a hallmark of resistance to MAPKi in melanoma. Consistent with this, many genes of cluster 1 belong to metabolic signatures. As a matter of fact, the use of OXPHOS and fatty acid inhibitors (see previous paragraph) potentiate MAPKi bypassing the dynamics between fast cycling and slow cycling phenotypes. However, two important limitations emerged: (1) low concordance in the molecular targets among studies and (2) results obtained only at single drug level. To overcome this issue, a combined approach of mitochondrial inhibitors together with inhibitors of MUFAs and FAO could be a successful strategy.

Very recently, it has been reported that the inhibition of the fatty acid transporter FATP2 using the specific inhibitor lipofermata reduces the accumulation of lipids and also challenges the mitochondrial metabolism in an aged melanoma microenvironment. This allows the overcoming of age-related resistance to MAPKi in melanoma mouse models [89]. This study paves the way to additional combinatorial strategies using FATP2 inhibitors together with inhibitors of enzymes involved in MUFAs synthesis, such as SCD itself.

Metabolic inhibitors may also be combined with additional druggable targets belonging to cluster 2, such as NFκB and mTOR. Interestingly, mitochondrial hyperfusion, a process that antagonizes apoptosis is an adaptive response to mTOR inhibition [90] and may be overcome by the combination with mitochondrial inhibitors. It is important to point out that while mTOR inhibitors, such as rapamycin analogs (i.e., rapalog), have been approved in the clinic to treat cancer, NFκB inhibitors efficacy as therapeutics is limited because of the host toxicity [91].

It is also worth considering the therapeutic opportunities provided by YAP/TAZ-TEAD inhibitors, many of which are currently under clinical development. These compounds are divided into three groups: (1) inhibitors of YAP/TAZ stimulators, (2) direct inhibitors of YAP/TAZ-TEAD and (3) drugs blocking the oncogenic downstream YAP/TAZ transcriptional target genes [92].

Finally, CSCs may also be directly tackled thanks to the use of specific inhibitors of JARID1B [93] despite in this case their clinical development being far from being successfully accomplished.

In summary, we believe that in order to cut the Gordian knot linking drug resistance with CSCs’ fitness in metastatic melanomas new therapeutic strategies will have to be rationally developed and take into account the simultaneous targeting of multiple nodes in the limited key pathways identified.

## 4. Methods

### 4.1. Bibliographic Search

A bibliographic search was conducted through “PubMed” free resource supporting the biomedical and life science research. Keywords used were: “melanoma stem cells”, “resistance to target therapy”, “metabolic rewiring” and “phenotype switching”. In this way we found more than 3000 publications. The list was refined according to the: (a) potential concordance in the genes identified among studies, (b) most recent works (c) highly indexed journals (impact factor >7) and (d) studies taking advantages of single-cell approaches. Thereby, a list of 25 top genes was obtained, namely “MSCsign”.

### 4.2. Interactomic and Clustering Plots

“MSCsign” was subjected to the Markov Cluster Algorithm [82] and four clusters identified: (1) kinase and metabolic, (2) melanoma-associated, (3) Hippo pathway and (4) slow cycling/CSCs. The same gene list was used to build a protein−protein interaction (PPI) network using the STRING online database [83]. The minimum required interaction score used was >0, 4, 89 hedges were identified. *p*-value < 1.0 × 10^−16^.

### 4.3. Mining TCGA Data of SKCM Dataset

The expression levels of the genes belonging to “MSCsign” were evaluated in 480 samples coming from the skin cutaneous melanoma dataset using UALCAN software [84]. Those analyses were refined according to the mutational status of melanoma patients extracting RNA-Seq data from skin cutaneous melanoma dataset (TCGA, PanCancer Atlas) [85] available on the cBioPortal website [86,87]. mRNA Expression, RSEM (RNA-seq by expectation-maximization) (batch normalized from Illumina HiSeq_RNASeqV2). Heatmaps wereplotted through the online tool Orange. Kaplan–Meyer curves were used to estimate the prognostic values of “MSCsign” for each mutational subset.

### 4.4. Principal Component Analyses

The expression levels of “MSCsign” from skin cutaneous melanoma dataset were subjected to Principal Component Analysis (PCA) in comparison with other six solid cancers through the online software GEPIA [88]. SKCM = Skin Cutaneous Melanoma, BRCA = Breast invasive carcinoma, LUAD = Lung adenocarcinoma, OV = Ovarian serous cystadenocarcinoma, COAD = colon adenocarcinoma, GBM = Glioblastoma multiforme, HNSC = Head and Neck squamous cell carcinoma.

### 4.5. Statistical Analyses

*p*-values were estimated using the log-rank test (significance *p* < 0.05).

### 4.6. List of the Online Tools Used

https://pubmed.ncbi.nlm.nih.gov/
https://string-db.org/
http://ualcan.path.uab.edu/index.html
https://www.cbioportal.org/
http://gepia.cancer-pku.cn/index.html
https://orange.biolab.si/

## Figures and Tables

**Table 1:** List of 25 top genes relevant for melanoma stem cells and resistance to target therapy.

Gene	Description	Reference #	Cluster
MAP2K1	Dual specificity mitogen-activated protein kinase kinase 1	[9,11,12,13,14,15]	**Kinase and metabolic signature**
JUN	Transcription factor AP-1	[15,42,46,56,58]	
RXRG	Retinoic acid receptor RXR-gamma	[42,53]	
CPT1A	Carnitine O-palmitoyltransferase 1	[59]	
AKT1	RAC-alpha serine/threonine-protein kinase	[9,16,20]	
BRAF	Serine/threonine-protein kinase B-raf	[4,7,9,11,12,13,14]	
PPARA	Peroxisome proliferator-activated receptor alpha	[59]	
ATF4	Cyclic AMP-dependent transcription factor ATF-4	[20,60]	
PPARGC1A	Peroxisome proliferator-activated receptor gamma coactivator 1-alpha	[20,61,62,63]	
SCD	Acyl-CoA desaturase	[60,64]	
TFAM	Transcription factor A, mitochondrial	[65]	
HIF1A	Hypoxia-inducible factor 1-alpha	[20,66]	
MITF	Microphthalmia-associated transcription factor	[20,42,46,47,50,52,53,56,57,60,61,62,64,67]	**Melanoma-associated signature**
MTOR	Serine/threonine-protein kinase mTOR	[62,67]	
SERPINE2	Glia-derived nexin	[68]	
AXL	Tyrosine-protein kinase receptor UFO	[20,42,46,47,50,52,53,56,57,58,60,66]	
MLANA	Melanoma antigen recognized by T-cells 1	[52,53,56,66]	
NFKB1	Nuclear factor NF-kappa-B p105 subunit	[52,66]	
SOX10	Transcription factor SOX-10	[53,58]	
TP53	Cellular tumor antigen p53	[69]	
YAP1	Transcriptional coactivator YAP1	[47,64]	**Hippo pathway signature**
TEAD1	Transcriptional enhancer factor TEF-1	[47,58]	
TAZ	Tafazzin	[47,64]	
NGFR	Tumor necrosis factor receptor superfamily member 16	[52,53,54,56,57,58,66,70,71]	**Slow cycling signature**
KDM5B	Lysine-specific demethylase 5B	[55,56,63,64,66,70,72]	

Bold: Genes divided into four major clusters.

## Supplementary Materials

The following are available online at https://www.mdpi.com/2072-6694/12/11/3368/s1. Table S1. Enrichment analyses of Gene Ontology, KEGG and Reactome on the list of 25 top genes performed using STRING online database. Table S2. Gene expression levels of “MSCsign” normalized considering the maximum median expression value coming from SKCM dataset. Table S3. Raw data of gene expression levels obtained from https://www.cbioportal.org/. Table S4. OS data obtained from https://www.cbioportal.org/.

## Abbreviations

MAPKi	BRAF/MEK inhibitors
CSCs	cancer stem cells
sc	Single Cell
OXPHOS	Oxidative Phosphorylation
MUFAs	Monounsaturated Fatty Acids
FAO	Fatty Acid Oxidation
PPI	Protein−Protein Interaction
MSCsign	melanoma stem cells signature
